# Genetic diversity and variation in antimicrobial-resistance determinants of non-serotype 2 *Streptococcus suis* isolates from healthy pigs

**DOI:** 10.1099/mgen.0.000882

**Published:** 2022-11-03

**Authors:** Nattinee Kittiwan, Jessica K. Calland, Evangelos Mourkas, Matthew D. Hitchings, Susan Murray, Pakpoom Tadee, Phacharaporn Tadee, Kwanjit Duangsonk, Guillaume Meric, Samuel K. Sheppard, Prapas Patchanee, Ben Pascoe

**Affiliations:** ^1^​ Department of Food Animal Clinics, Faculty of Veterinary Medicine, Chiang Mai University, Chiang Mai 50100, Thailand; ^2^​ Integrative Research Centre for Veterinary Preventive Medicine, Faculty of Veterinary Medicine, Chiang Mai University, Chiang Mai 50100, Thailand; ^3^​ Veterinary Research and Development Center (Upper Northern Region), Hang Chat, Lampang 52190, Thailand; ^4^​ Oslo Centre for Biostatistics and Epidemiology, Oslo University Hospital, Oslo, Norway; ^5^​ Ineos Oxford Institute for Antimicrobial Research, Department of Biology, University of Oxford, South Parks Road, Oxford, UK; ^6^​ Swansea University Medical School, Swansea University, Singleton Park, Swansea, UK; ^7^​ Faculty of Animal Science and Technology, Maejo University, Chiang Mai 50290, Thailand; ^8^​ Department of Microbiology, Faculty of Medicine, Chiang Mai University, Chiang Mai 50200, Thailand; ^9^​ Milner Centre for Evolution, Department of Biology and Biochemistry, University of Bath, Claverton Down, Bath, UK; ^10^​ Faculty of Allied Medical Science, Chiang Mai University, Chiang Mai, 50200, Thailand; ^11^​ Centre for Genomic Pathogen Surveillance, Big Data Institute, University of Oxford, Old Road Campus, Oxford, UK; ^†^​ Present address: Pathogen Genomics Unit, Public Health Wales, Cardiff, Wales, UK; ^‡^​ Present address: Cambridge Baker Systems Genomics Initiative, Baker Heart and Diabetes Institute, Melbourne, Victoria, Australia

**Keywords:** antimicrobial resistance, gene pool transmission, meningitis, One Health, *Streptococcus suis*

## Abstract

*
Streptococcus suis* is a leading cause of bacterial meningitis in South-East Asia, with frequent zoonotic transfer to humans associated with close contact with pigs. A small number of invasive lineages are responsible for endemic infection in the swine industry, causing considerable global economic losses. A lack of surveillance and a rising trend in clinical treatment failure has raised concerns of growing antimicrobial resistance (AMR) among invasive *S. suis *. Gene flow between healthy and disease isolates is poorly understood and, in this study, we sample and sequence a collection of isolates predominantly from healthy pigs in Chiang Mai province, Northern Thailand. Pangenome characterization identified extensive genetic diversity and frequent AMR carriage in isolates from healthy pigs. Multiple AMR genes were identified, conferring resistance to aminoglycosides, lincosamides, tetracycline and macrolides. All isolates were non-susceptible to three or more different antimicrobial classes, and 75 % of non-serotype 2 isolates were non-susceptible to six or more classes (compared to 37.5 % of serotype 2 isolates). AMR genes were found on integrative and conjugative elements previously observed in other species, suggesting a mobile gene pool that can be accessed by invasive disease isolates. This article contains data hosted by Microreact.

## Data Summary

Short read data are available from the NCBI (National Center for Biotechnology Information) SRA (Sequence Read Archive), associated with BioProject PRJNA418954 (https://www.ncbi.nlm.nih.gov/bioproject/PRJNA418954). Assembled genomes and supplementary material are available from FigShare: doi: 10.6084/m9.figshare.13385465 [1].

**Impact Statement**

The zoonotic pathogen *Streptococcus suis* causes respiratory and systemic disease in pigs and is among the most common causative agents of human clinical bacterial meningitis in South-East Asia, particularly in China, Thailand and Vietnam. We collected isolates from healthy farmed pigs in Northern Thailand, representing a source population from which invasive isolates have recently emerged. Pangenome characterization of the isolates revealed increased genetic diversity and antimicrobial resistance (AMR), suggesting that One Health approaches may be beneficial in tackling the increase in AMR.

## Introduction

More than half of the world’s pork meat is produced in South-East Asia, and China alone is home to nearly half of the world’s livestock pigs. Technological improvements contributed to commercialization of pork production in the early part of the 20th century and the numbers of farmed swine rapidly expanded from ~400 million (in 1961) to an estimated 1 billion swine reared for meat in 2018 [2]. This massive increase in agricultural intensification has brought significant challenges in animal welfare, including infection control. Among the most common infections to Asian herds is a systemic disease caused by *Streptococcus suis* [3]. Initial infection through the nasopharynx can lead to septicaemia with arthritis, endocarditis, meningitis and sudden death among the symptoms [4–6]. *S. suis* infections accounted for a loss of over US$11 million (£10 million; £1=$1.1) to the pork industry in Thailand alone in 2019 [7]. This expanded niche for *S. suis* has provided opportunities for zoonotic infections, which are frequently reported worldwide following increased exposure to pigs, often in farm workers, slaughterhouse workers and butchers [6, 8, 9]. However, in South-East Asia, particularly in Thailand, Vietnam and China where there is a tradition of consuming raw pork dishes, *S. suis* infection is one of the most common causative agents of clinical bacterial meningitis [10, 11].

Human zoonotic *S. suis* infections predominantly arise from a single virulent lineage, thought to have first emerged in the 1920s alongside the intensification of the pork production industry. However, no consistent genome differences between pig and human disease isolates have been observed [12]. This may be related to the fact that isolates from healthy (asymptomatic) pigs have not been well studied. It has been observed that disease-associated isolates have fewer genes overall (smaller genomes), but more genes that encode putative virulence factors [13, 14]. Serotyping of the *S. suis* capsular polysaccharides is often used in epidemiological studies, with 29 *S *. suis* sensu stricto* serotypes described to date [15, 16]. *S. suis* serotype 2 is the most virulent and is frequently isolated from diseased pigs and human clinical cases [17, 18]; however, non-serotype 2 isolates (often isolated from healthy pigs) represent an extensive reservoir of genetic diversity [19–22].

Widespread use of antimicrobial drugs in the pig production industry has driven an increase in antimicrobial resistance (AMR) [23, 24]. Imprudent use of colistin in pork production as a growth enhancer (since the 1970s) encouraged the development of resistance in *Escherichia coli* (and other Gram-negative bacteria), which has diminished the effectiveness of antibiotics used in human medicine [25–27]. Furthermore, there is a rising trend in multi-drug resistant zoonotic pathogens, which pose a significant public-health threat [28–30]. Regulation of veterinary use of antibiotics is difficult in low- and middle-income countries, which consequently have some of the highest AMR levels in the world [31]. For example, in Thailand alone, infections with antimicrobial-resistant bacteria are estimated to cause up to 38 000 human deaths each year [32]. A lack of surveillance and a rise in clinical treatment failure has raised concerns of growing AMR among invasive *S. suis* [33].

Most studies have focussed on a few important *S. suis* clones that are responsible for the majority of human infection cases, such as the serotype 2 (most common in Asia) and serotype 9 (most common in Europe) groups. Given the frequent homologous recombination between *Streptococcus* species, and following a One Health approach to disease surveillance, we aimed to characterize the genetic diversity of non-serotype 2 *S *. *suis* isolates in this study. We sampled and sequenced a collection of isolates from healthy pigs in Chiang Mai province, Northern Thailand. Pangenome comparisons with a selection of archived, invasive serotype 2 isolates identified increased genetic diversity and more frequent AMR carriage in isolates from healthy pigs. AMR genes were found on integrative and conjugative elements (ICEs) previously observed in other species, suggesting a mobile gene pool that can be accessed by invasive disease-causing isolates.

## Methods

### Ethics

This study was carried out according to guidelines for the care and use of laboratory animals [34].

### Sample collection

Samples were collected between March and November 2015, with a total of 760 tonsil swab samples collected from 111 pig farms in 25 districts of Chiang Mai province, Thailand. All swab samples were kept in Stuart transport medium (Oxoid) and transported to the laboratory at 4 °C within 24 h of collection. Livestock pigs were swabbed, and *S. suis* identified in 138 samples (18.2 %). Of the 138 *S *. *suis* isolates obtained from healthy pigs, only 1 isolate (0.7 %) was confirmed as *S. suis* serotype 9. Meanwhile, all the remaining 137 isolates (99.3 %) were negative to serotypes 1/2, 1, 2, 7, 9 and 14 by PCR identification and classified as non-serotype 2 strains. Among 138 strains, 25 strains were randomly selected for whole-genome sequencing (WGS). In addition, 11 isolates were selected from laboratory archives and sequenced for comparison. These included additional non-serotype isolates from healthy pigs (*n*=3) collected at an earlier date, from different farms; and serotype 2 isolates from healthy pigs (*n*=4) and infected pigs (*n*=2) submitted to the Veterinary Research and Development Center (Upper Northern Region), Thailand, collected between 2010 and 2013. Finally, two serotype 2 clinical isolates (*n*=2) were also included for comparison – cultured from the blood of meningitis patients collected at the Faculty of Medicine, Chiang Mai University, Thailand (in 2010).

### Bacterial identification and growth

Tonsil swab samples were inoculated onto 5 % sheep blood agar plates (Oxoid) and incubated at 37 °C for 24 h. *S. suis* isolates were identified by biochemical characterization [35], and small (approximately 1 mm in diameter) transparent α-haemolysis and non-haemolysis colonies of Gram-positive cocci with negative catalase test were selected for further screening. Criteria for presumptive identification of *S. suis* included no growth on 6.5 % NaCl agar, a negative Voges-Proskauer (VP) test, and production of acid in trehalose, lactose, sucrose, salicin and inulin broths, but no acid production in glycerol, sorbitol and mannitol. A multiplex PCR using primers specific to the 16S rRNA gene was used to confirm the identification of *S. suis* and capsular gene types 1 or 14, 2 or 1/2, 7, and 9, which are the most prevalent serotypes recovered from diseased pigs, as described in Table S1 (available with the online version of this article) [36–38].

### Antimicrobial-susceptibility testing

Antimicrobial-susceptibility tests were performed using the disc diffusion method in accordance with the recommendations of the Clinical and Laboratory Standards Institute (CLSI) [39]. Eighteen antibiotic drugs from nine antibiotic groups were tested, including aminoglycoside (gentamicin, 10 µg; and kanamycin, 30 µg), lincosamides (lincomycin, 10 µg; and clindamycin, 2 µg), macrolides (erythromycin, 15 µg), tetracyclines (tetracycline, 30 µg; doxycycline, 30 µg; and oxytetracycline, 30 µg), oxazolidinone (linezolid, 30 µg), phenicols (chloramphenicol, 30 µg; and florfenicol 30 µg), β-lactams (ampicillin, 10 µg; penicillin G, 10 units; amoxicillin, 10 µg; amoxicillin/clavulanic acid, 30 µg; and ceftiofur, 30 µg), fluoroquinolones (enrofloxacin, 5 µg) and folate inhibitors (sulfamethoxazole/trimethoprim, 1.25/23.75 µg) (Oxoid). *Streptococcus pneumoniae* ATCC 49619 was used as a positive control and diameter breakpoints were assessed according to the guidelines described in Table 1 [39–46]. Fisher’s exact tests were performed using SPSS Statistics version 22 (IBM) to determine the difference of antimicrobial susceptibility between *S. suis* serotype 2 and non-serotype 2 isolates. The association between AMR phenotype and genotype was tested by Fisher’s exact test. Statistically significant associations were shown as odds ratios (ORs) with 95 % confidence intervals (CIs). Results were considered statistically significant when a two-tailed *P* value was ≤0.05.

**Table 1.** Antimicrobial-susceptibility test results by disc diffusion method of 36 *S.suis*, grouped by serotype Susceptible (S), intermediate (I) and resistant (R) phenotypes are indicated. An asterisk (*) indicates statistical significance by Fisher's exact test; *P* value < 0.05.

Antibiotic agent	Zone of inhibition (mm)			* S. suis* (%) (*n*=36)			Serotype 2 (%) (*n*=8)			Non-serotype 2 (%) (*n*=28)			*P* value
	S	I	R	S	I	R	S	I	R	S	I	R	
GEN†	≥16	13–15	≤12	30.6	38.8	30.6	0	37.5	62.5	39.3	39.3	21.4	0.076
KAN‡	≥18	14–17	≤13	0	11.1	88.9	0	25	75	0	7.1	92.9	>0.999
LIN§	≥19	16–18	≤15	0	0	100	0	0	100	0	0	100	>0.999
CLI||	≥19	16–18	≤15	2.8	0	97.2	12.5	0	87.5	0	0	100	0.222
ERY||	≥21	16–20	≤15	16.7	13.9	69.4	12.5	0	87.5	17.8	14.3	67.9	>0.999
TET||	≥28	25–27	≤24	2.8	5.6	91.7	0	0	100	3.6	7.1	89.3	>0.999
DOX||	≥28	25–27	≤24	0	8.3	91.7	0	0	100	0	10.7	89.3	>0.999
OTC§	≥26	16–25	≤15	5.6	11.1	83.3	0	0	100	7.1	14.3	78.6	>0.999
LZD*||*	≥21	–	–	100	0	0	100	0	0	100	0	0	>0.999
CHL*||*	≥21	18–20	≤17	44.4	47.2	8.3	0	100	0	57.1	32.1	10.7	0.005*
FLO¶	≥22	19–21	≤18	72.2	22.2	5.6	75	25	0	71.4	21.4	7.1	>0.999
AMP†	≥24	23–17	≤16	80.6	13.9	5.6	100	0	0	75	17.9	7.1	0.309
PEN‡	≥26	13–25	≤12	30.6	66.7	2.8	87.5	12.5	0	14.3	82.1	3.6	0.001*
AMX#	≥24	15–23	≤14	83.3	13.9	2.8	100	0	0	78.6	17.9	3.6	0.302
AMC††	≥18	14–17	≤13	97.2	2.8	0	100	0	0	96.4	3.6	0	>0.999
CTF*¶*	≥21	18–20	≤17	94.4	5.6	0	100	0	0	92.9	7.1	0	>0.999
ENR‡	≥23	19–22	≤18	58.3	30.6	11.1	62.5	37.5	0	57.1	28.6	14.3	>0.999
SXT*||*	≥19	16–18	≤15	72.2	5.6	22.2	87.5	0	12.5	67.9	7.1	25	0.397

Interpretative criteria according to: †CLSI 2017; ‡EUCAST (European Committee on Antimicrobial Susceptibility Testing) and CLSI 2013; §CLSI 2008; ||CLSI 2020; ¶CLSI 2018; #Howe and Andrews 2012 [46]; and ††CLSI 2002 guidelines.

### Genome sequencing and assembly

Twenty-five *S. suis* isolates from pigs with no clinical signs of *S. suis* infection (healthy pigs) were randomly selected for sequencing from the 138 recovered samples. Our collection was augmented with two archived isolates derived from tissue samples of pigs with clinical signs of *S. suis* infection (diseased pigs) that were submitted to the Veterinary Research and Development Center (Upper Northern Region) of the National Institute of Animal Health (Thailand), and a further nine isolates from the Faculty of Medicine at Chiang Mai University (Thailand). In total, our collection included 32 healthy pigs, 2 diseased pigs and 2 human clinical samples cultured from the blood of meningitis patients. All strains were cultured in Todd–Hewitt-broth at 37 °C for 18–24 h, and genomic DNA was extracted using the QIAamp DNA minikit (QIAgen). WGS using a multiplex sequencing approach was performed on an MiSeq genome sequencer (Illumina) using Nextera XT libraries and third-generation MiSeq reagent kits. Paired-end short reads of 300 bp were filtered, trimmed and assembled *de novo* with SPAdes version 3.7 [47], using the *-careful* command. The mean number of contiguous sequences (contigs) in 36 *S *. *suis* genomes was 160, for a mean total assembled sequence size of 2.22 Mbp. The mean N50 contig length (L50) was 66 810 and the mean G+C content was 41.3 mol%. Short-read data are available in the NCBI (National Center for Biotechnology Information) SRA (Sequence Read Archive), associated with BioProject PRJNA418954. Assembled genomes and supplementary material are available from FigShare (10.6084 /m9.figshare.13385465; individual accession numbers and assembled genome statistics are in Table S2).

### Population structure and phylogeny

Isolate genomes were uploaded to pubMLST and sequence typed *in silico*, according to the seven gene multilocus sequence typing (MLST) scheme [48]. Many of our non-serotype 2 isolate genomes could not be typed and were allocated novel MLST sequence types (27 of 36; Table S2); two isolates remained unassigned to a sequence type. A multisequence alignment was created from concatenated gene sequences of all core genes (found in >95 % isolates) from the reference genome, BM407 [49], using mafft [50] on a gene-by-gene basis [51] (size 1 202 840 bp; FigShare: doi: 10.6084 /m9.figshare.13385465). Maximum-likelihood phylogenies were reconstructed with iq-tree (version 1.6.8) using the GTR+F+I+G4 substitution model and ultra-fast bootstrapping (1 000 bootstraps) [52]; and visualized on Microreact [53]: https://microreact.org/project/Ssuis-ns2.

### Accessory genome characterization

All unique genes present in at least one isolate (the pangenome) were identified by automated annotation using prokka (version 1.13; default parameters) followed by pirate (version 1.0.4; default parameters), a pangenome tool that allows for orthologue gene clustering in bacteria [54, 55]. We defined genes in pirate using a wide range of amino acid percentage sequence identity thresholds for Markov cluster algorithm clustering [46, 51, 56–61]. The pangenome of all 36 isolates contained 5 004 genes, of which 1 348 genes were shared by all isolates (>95 %) and defined the core genome (Table S3). Pairwise core and accessory genome distances were compared using PopPUNK (version 1.1.4; using the *-easy run* option) [62], which uses pairwise nucleotide *k*mer comparisons to distinguish shared sequence and gene content to identify accessory genome divergence in relation to the core genome. A two-component Gaussian mixture model was used to build a network to define clusters (components, 41; density, 0.0579; transitivity, 0.9518; score, 0.8967).

### Identification of AMR, virulence and plasmid genes

The accessory genome of each isolate was characterized, including detection of putative virulence factors, AMR genes, and known plasmid genes using abricate (version 0.9.8) (https://github.com/tseemann/abricate) and the VFDB (Virulence Factor Database), NCBI AMRfinder Plus, CARD (Comprehensive Antibiotic Resistance Database), ResFinder, and PlasmidFinder databases (10th September 2019 update; Tables S4–S6) [56, 63–68]. Results were similar between all three AMR databases, and we report results from the curated NCBI AMRfinder Plus database of 1 726 resistance genes covering 15 antimicrobial agent types; including genes associated with resistance to aminoglycosides, β-lactams, colistin, fluoroquinolones, fosfomycin, fusidic acid, glycopeptides, MLS_B_ (macrolide-lincosamide-streptogramin B), nitroimidazole, oxazolidinones, phenicols, rifampicin, sulphonamides, tetracyclines and trimethoprim. A threshold of 70 % identity, over 50 % gene length (default settings) was used for reporting a match between a gene in the NCBI AMRfinder Plus database and the input genome. ICEs were detected using nucleotide comparisons with known ICEs in the PlasmidFinder and mob-suite databases. Individual mobile genes were identified with PlasmidFinder, while mob-suite (version 2.1.0; default parameters) compares sequences to all plasmid sequences in the NCBI repository (Table S7).

### Asian context collection

For greater context, all Asian genomes shared publicly on the pubMLST *S. suis* database (*n*=112) were downloaded for comparison with our dataset. Genomes were predominantly of Chinese origin (*n*=96), with some additional genomes from Thailand (*n*=14). A phylogeny was reconstructed for these genomes alongside out collection using iq-tree, as previously described for our collected isolates. All genomes were screened for AMR genes using NCBI’s AMRfinder Plus (Table S8).

## Results

### Samples

**Fig. 1.**
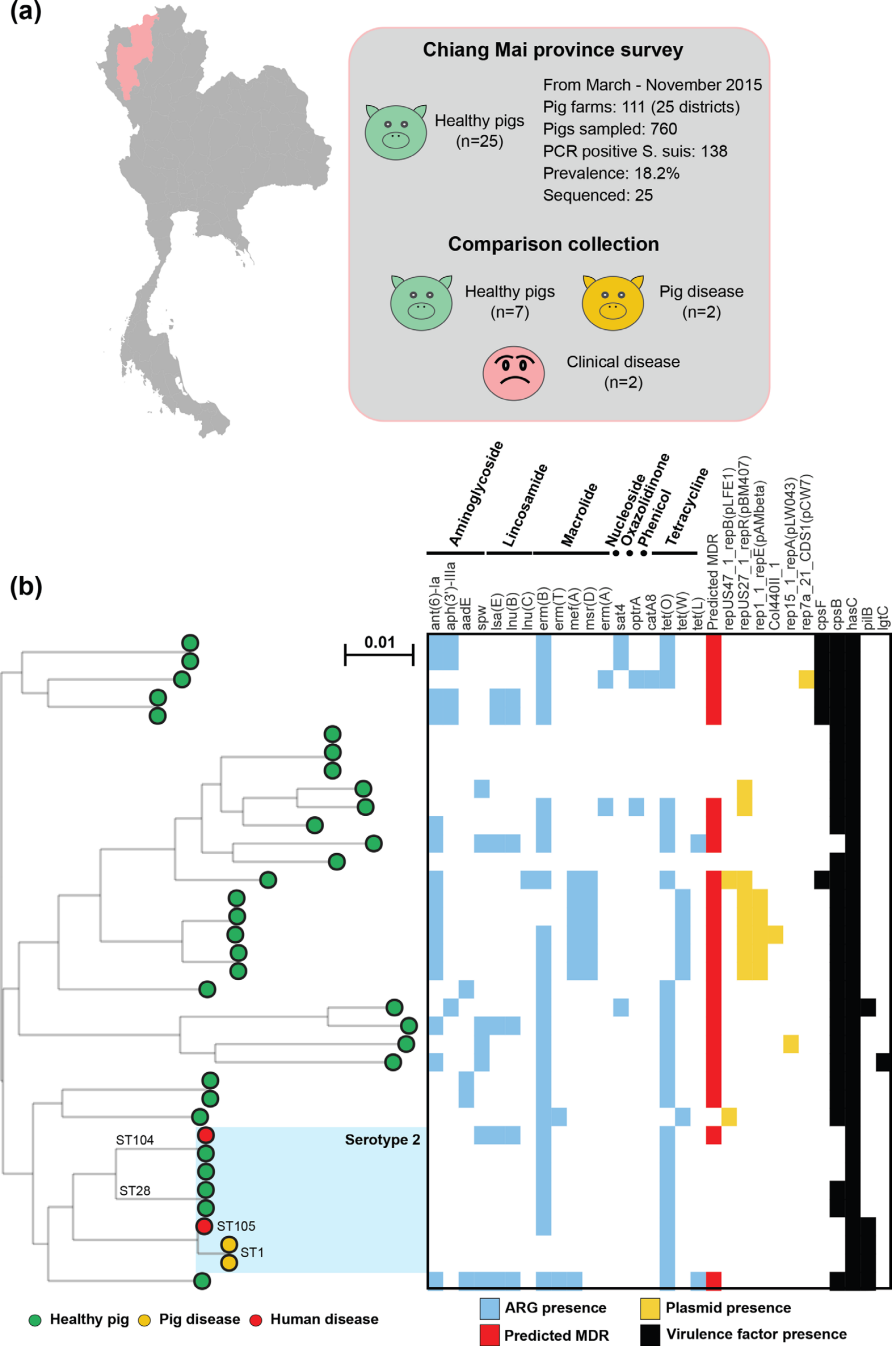
(a) Isolates were collected as part of a survey of healthy pigs in Chiang Mai province, Thailand. (**b)** Population structure of selected sequenced isolates compared with other serotype 2 genomes from the same region. All core genes (present in ≥95 % of isolates) from the reference genome (1348 genes) were used to build a gene-by-gene alignment (*n*=36; 1 202 840 bp). A maximum-likelihood phylogeny was reconstructed with iq-tree, using a GTR model and ultrafast bootstrapping (1000 bootstraps; version 1.6.8) [52, 120]. Bar, genetic distance of 0.01. Leaves are coloured by disease status and host: samples from healthy pigs are green; diseased pigs are yellow; and samples from human clinical cases are red. Serotype 2 isolates are shaded in blue, with common sequence types annotated. The presence of AMR genes, known plasmids and virulence genes identified using abricate and NCBI, PlasmidFinder and VFDB (Virulence Factor Database) are indicated by coloured blocks. Interactive visualization is available on Microreact: https://microreact.org/project/Ssuis-ns2 [53].

All *S. suis* samples collected from healthy pigs in Chiang Mai province in Thailand were identified by PCR (Table S1) as non-serotype 2 isolates. From the 138 isolates we collected, 25 were randomly selected for WGS. An additional 11 isolates from laboratory archives, previously collected from Chiang Mai, were added to the dataset to include representative isolates from pig disease and invasive human infection. In total, the dataset used consisted of 36 isolates, of which 8 isolates (22.2 %) were serotype 2, including isolates from human clinical cases (*n*=2), diseased pigs (*n*=2) and healthy pigs (*n*=4), and 28 isolates (77.8 %) of non-serotype 2 *S *. *suis* from healthy pigs (Fig. 1a, Table S2).

### Core and accessory genome characterization

**Fig. 2.**
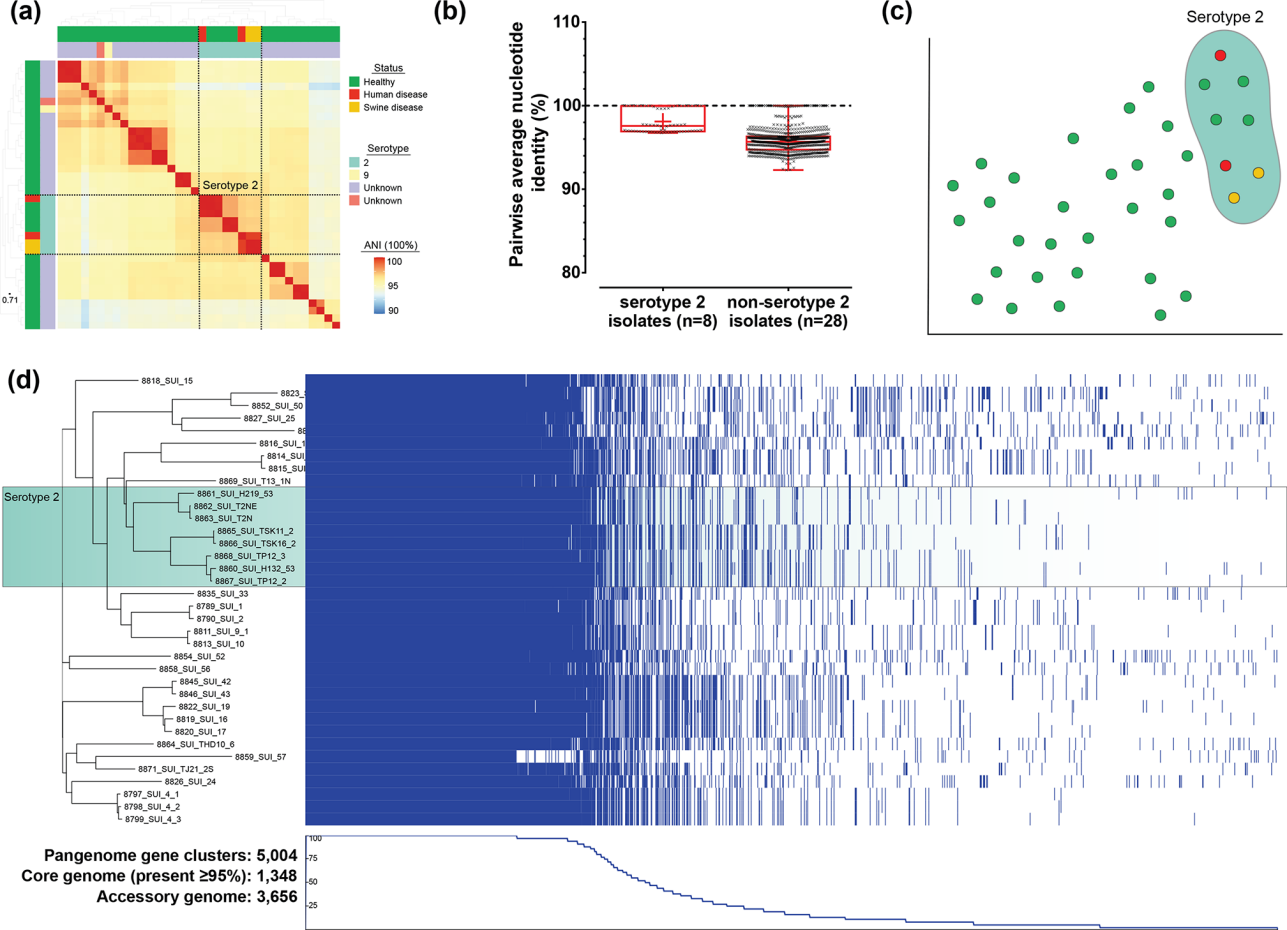
(a) Heatmap of pairwise average nucleotide identity (ANI). Highly similar pairwise comparisons are coloured in red to blue for the most dissimilar isolates. The cluster of serotype 2 isolates is boxed. (**b)** Summary of pairwise comparisons between serotype 2 and non-serotype 2 isolates. (**c)** PopPUNK pairwise accessory distances visualized with t-distributed stochastic neighbor embedding (t-SNE) clustering in microreact: https://microreact.org/project/Ssuis-ns2 [53]. (**d)** Visualization of the pangenome (pirate) with Phandango, including estimation of the core (gene present in 95 % or more isolates) and accessory genome composition [55, 121].

Non-serotype 2 isolates were not responsible for disease in either pigs or humans. A maximum-likelihood phylogeny reconstructed from a concatenated gene-by-gene core genome alignment (1348 genes) revealed a highly structured population (Fig. 1b). Serotype 2 isolates clustered together, including the previously described sequence types ST-1, ST-28, ST-104, ST-105 and a novel sequence type designated ST-1939. Non-serotype 2 isolates clustered into 17 sequence types, all of which were novel sequence types uploaded to pubMLST (Table S2). Pairwise average nucleotide identity comparisons suggested that non-serotype 2 isolates (75.1 % identical) were more diverse than serotype 2 isolates (98.1 % identical) in the core genome (Fig. 2a,b). This was supported by (pairwise) clustering of the core and accessory genome using PopPUNK [62], which identified divergence in the accessory genomes of the serotype 2 isolates (Fig. 2c). Together, the pangenome of all 36 isolates comprised 5004 gene clusters, with 1348 core genes present in at least 95 % of isolates representing ~27 % of the pangenome; or ~68 % of the average *S. suis* genome (1993 ORFs in BM407; Fig. 2d, Table S3). Typically, invasive serotype 2 isolates have smaller genomes but contain more virulence-related genes [12]. In our dataset, this was also true with serotype 2 isolates having smaller genomes on average (Table S2), and the virulence associated *pilB* gene was found in 75 % (*n*=3 of 4) of invasive isolates, but only 7 % of isolates from healthy pigs (*n*=2 of 28) (Fig. 1b, Table S4).

### Widespread AMR determinants in *S. suis* isolates from healthy pigs

We scanned all 36 genomes for known determinants of AMR through nucleotide comparisons (≥70 % sequence identity, over 50 % gene length) with three AMR gene databases (NCBI AMRfinder Plus, CARD and Resfinder) [56, 64–66, 68]. Results were similar between all databases, and we report results from the curated AMRfinder database, where we identified 18 resistance genes from seven different antimicrobial classes (Fig. 1b, Table S5). Loci conferring putative resistance to aminoglycosides [*aadE*, *ant(6)*-*Ia*, *aph*(*3’*)-*III* and *spw*], macrolides [*erm*(*A*)*, erm*(*B*)*, erm*(*T*)*, mef*(*A*) and *msr*(*D*)], lincosamides [*lsa(E*), *lnu*(*B*) and *lnu*(*C*)], tetracyclines [*tet(W*), *tet*(*L*) and *tet*(*O*)], oxazolidinone (*optrA*), nucleoside (*sat4*) and chloramphenicol (*catA8*) were found in 32 isolates (89 %). On average, fewer antibiotic-resistance genes were identified in the serotype 2 isolates (five genes) compared to non-serotype 2 isolates (18 genes; Table S5). All 18 of the resistance genes were detected in the non-serotype 2 isolates from healthy pigs, but only 5 of the potential AMR genes *spw*, *lsa(E)*, *erm*(*B*), *lnu*(*B*) and *tet*(*O*) were found in the eight serotype 2 isolates, including *tet*(*O*), which was present in all serotype 2 isolates. At least one AMR gene from three or more antimicrobial classes was found in 21 out of 36 isolates (58 %), and only one out of these was a *S. suis* serotype 2 isolate (id8860_SUI_H132_53).

### Evidence of mobility of AMR genes among *S. suis* from healthy pigs

Comparison of nucleotide sequences from all the genomes with the PlasmidFinder database [67] identified loci on six putative ICEs, including pLFE1, pBM407, pAMbeta, Col440II, pLW043 and pCW7 (Fig. 1b, Table S6). All putative ICEs were identified in non-serotype 2 isolates (39 %; 11 of 28). Two of these ICEs have previously been characterized in invasive *S. suis *, pBM407 (accession no. FM252033) and pAMbeta (accession no. AE002565.1). The pBM407 plasmid described in *S. suis* contained AMR genes conferring resistance to tetracycline [*tet(O), tet(L*)], chloramphenicol (acetyltransferase), erythromycin [*erm(B*)] and a dihydrofolate reductase [49]. However, plasmids from two different isolates with variation in gene content hint at an underlying diversity – and this potential composite architecture was evidenced in our study by differences in the AMR gene complement [49]. All serotype 2 isolates contain the *tetO* locus, and 75 % (6 of 8) contain the *ermB* locus, which are often found on pBM407 ICEs, but no other pBM407 genes were identified by this method (Table S6). Additional plasmids not previously described in *S. suis* were also identified using mob-suite, which compares genome sequences with all described plasmids in the NCBI database (Table S7) [69, 70].

### Widespread AMR in non-serotype 2 isolates

**Fig. 3.**
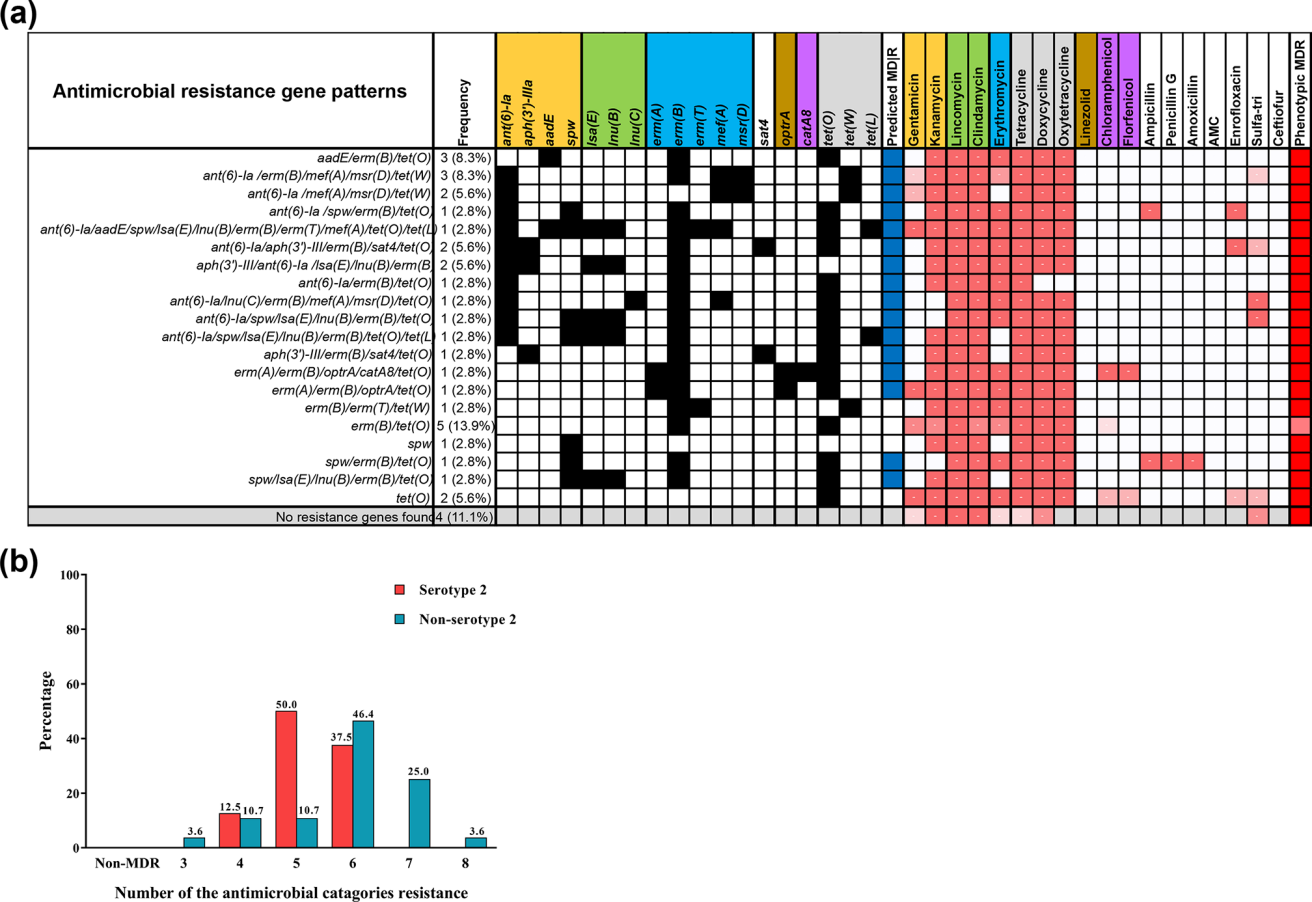
(a) Distribution of AMR patterns (black blocks indicate presence of AMR genes) summarized alongside their corresponding phenotypic resistance outcomes (red blocks indicate phenotypic resistance). Predicted (blue) and phenotypic (red) MDR is also indicated. (**b)** Summary of the number of different antimicrobial classes to which each isolate demonstrated phenotypic resistance. Isolates resistant to three or more different antimicrobial classes were characterized as having MDR. AMC, amoxicillin/clavulanic acid.

Disc diffusion assays were used to determine antimicrobial susceptibility of the isolates to 18 antimicrobial agents, from nine antimicrobial categories. Most isolates were highly susceptible to linezolid (100 %; *n*=36), amoxicillin/clavulanic acid (97 %; *n*=35), ceftiofur (94 %; *n*=34), amoxicillin (83 %; *n*=30) and ampicillin (81 %; *n*=29). High levels of resistance were observed against lincomycin (100 %; *n*=36), clindamycin (97 %; *n*=35), tetracycline (92 %; *n*=33), doxycycline (92 %; *n*=33), kanamycin (89 %; *n*=32), oxytetracycline (83 %; *n*=30), erythromycin (69 %; *n*=25) and gentamicin (31 %; *n*=11) (Table 1). Despite relatively low numbers of isolates, there was a statistically significant difference in antimicrobial susceptibility between *S. suis* serotype 2 and non-serotype 2 isolates for chloramphenicol (*P* value 0.005) and penicillin G (*P* value 0.001) using Fisher’s exact test (Table 1). Multi-drug resistance (MDR) is defined as an isolate that is non-susceptible to at least one antimicrobial agent from three different antimicrobial categories [71]. All 36 *S *. *suis* isolates were non-susceptible to three or more antibiotic classes (Fig. 3a, Table 1). Most (87.5 %; 7 of 8) serotype 2 isolates were non-susceptible to four and five antimicrobial categories; while three quarters (21 of 28) of non-serotype 2 isolates were non-susceptible to six, seven and eight antimicrobial categories (46.4, 25 and 3.6 %, respectively; Fig. 3b).

### Diverse AMR profiles in *S. suis* isolates from healthy pigs

Increased diversity in the core, accessory and plasmid content of non-serotype 2 isolates was associated with increased AMR conferred by 21 different AMR gene profiles (A–U; Fig. 3a, Table 2). The most common AMR gene pattern included the *erm*(*B*) and *tet*(*O*) genes (13.9 %), followed by *aadE*/*erm*(*B*)/*tet*(*O*) and *ant(6)*-*Ia/erm*(*B*)/*mef*(*A*)/*msr*(*D*)/*tet(W*) gene patterns (8.3 %). All putative resistance genes were absent in four non-serotype 2 strains (11.1 %). Non-serotype 2 isolates demonstrated greater variation in AMR gene content, with only three resistance gene patterns (B, C and P) found exclusively in serotype 2 isolates.

**Table 2.** AMR gene patterns of 36 *S. suis* isolates

Pattern	AMR gene pattern	Strain	Frequency
A	*spw*	FH57	1 (2.8 %)
B	*tet(O*)	DP-T2NE*^,^†, DP-T2N*^,^†	2 (5.6 %)
C	*erm(B)/tet(O*)	H219-53†,‡**,**TSK11-2†**,** TSK16- 2†**,** TP12-2†, TP12-3†	5 (13.9 %)
D	*aadE/erm(B)/tet(O*)	FH11, FH12, FH52	3 (8.3 %)
E	*ant(6)-Ia/erm(B)/tet(O*)	THD10-6	1 (2.8 %)
F	*erm(B)/erm(T)/tet(W*)	FH13	1 (2.8 %)
G	*spw/erm(B)/tet(O*)	FH25	1 (2.8 %)
H	*aph(3')-IIIa/erm(B)/sat4/tet(O*)	FH20	1 (2.8 %)
I	*ant(6)-Ia/spw/erm(B)/tet(O*)	FH51	1 (2.8 %)
J	*ant(6)-Ia/mef(A)/msr(D)/tet(W*)	FH16, FH17	2 (5.6 %)
K	*erm(A)/erm(B)/optrA/tet(O*)	TJ21-2S	1 (2.8 %)
L	*aph(3')-IIIa/ant(6)-Ia/erm(B)/sat4/tet(O*)	FH9, FH10	2 (5.6 %)
M	*aph(3')-IIIa/ant(6)-Ia/lsa(E)/lnu(B)/erm(B*)	FH1, FH2	2 (5.6 %)
N	*ant(6)-Ia/erm(B)/mef(A)/msr(D)/tet(W*)	FH19, FH42, FH43	3 (8.3 %)
O	*erm(A)/erm(B)/optrA/catA8/tet(O*)	FH33	1 (2.8 %)
P	*spw/lsa(E)/lnu(B)/erm(B)/tet(O*)	H132-53†,‡	1 (2.8 %)
Q	*ant(6)-Ia/lnu(C)/erm(B)/mef(A)/mrd(D)/tet(O*)	FH24	1 (2.8 %)
R	*ant(6)-Ia/lsa(E)/lnu(B)/erm(B)/tet(O)/tet(L*)	FH15§	1 (2.8 %)
S	*ant(6)-Ia/spw/lsa(E)/lnu(B)/erm(B)/tet(O*)	FH50	1 (2.8 %)
T	*ant(6)-Ia/aadE/spw/lsa(E)/lnu(B)/erm(B)/erm(T)/mef(A)/tet(O)/tet(L*)	T13-1N	1 (2.8 %)
U	No resistance genes found	FH4-1, FH4-2, FH4-3, FH56	4 (11.1 %)
**Total**			**36** (100 %)

*S. suis from diseased pigs.

†*S. suis* serotype 2.

‡*S. suis* from the human case.

§*S. suis* serotype 9.

### Phenotypic and genotypic concordance in AMR

**Fig. 4.**
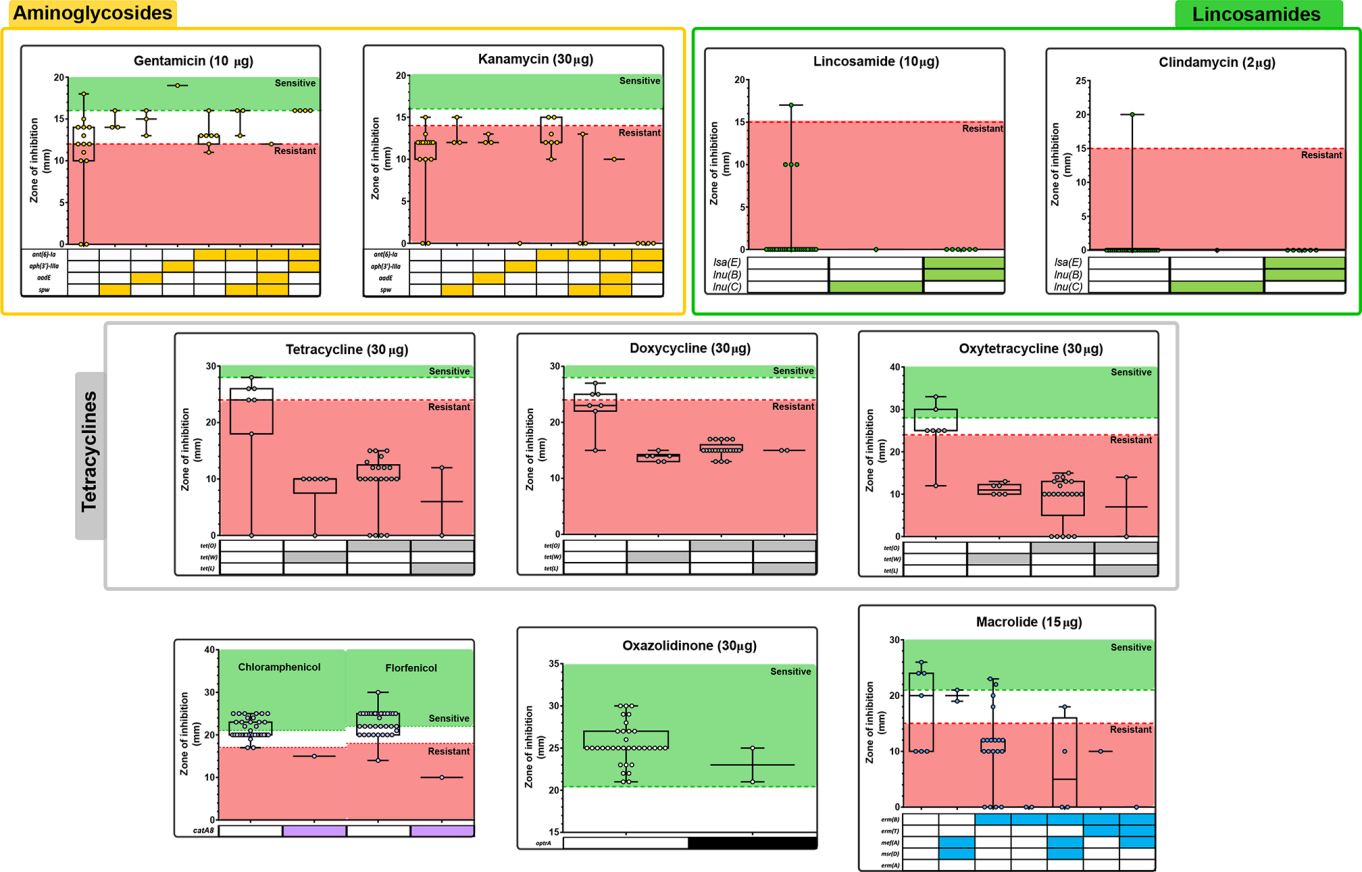
The effect of each AMR gene on phenotypic resistance diffusion diameters for aminoglycosides, lincosamides, tetracyclines, phenicols, oxazolidinone and macrolide. Zones of inhibition are shown for each AMR profile found in our dataset. Clinical breakpoints of resistance are indicated in red.

When we compared phenotypic non-susceptibility (zones of inhibition) with the presence of specific AMR genes, often there was no clear correlation (Table 3, Fig. 4). The strongest link between phenotype and AMR gene was observed for erythromycin resistance and the presence of *erm(B*) (OR=32.5, *P*=0.002), and 72.2 % of erythromycin-resistant isolates contained the *erm(B)* gene (Table 3). There were not enough resistant isolates to properly assess the correlation between the presence of genes linked to resistance to chloramphenicol (*n*=1) and florfenicol (*n*=1), and none of the isolates were resistant to the first-generation oxazolidinone, linezolid, despite identification of the corresponding *optrA* resistance gene (Table 3). The presence of AMR determinants did not equally contribute to resistance. In many cases, the accumulation of AMR genes provided increased resistance (and smaller zones of inhibition). Accumulation of tetracycline and macrolide determinants led to more extensive resistance profiles (Fig. 4). Although not identified in this study, point mutations will also play a significant role in conferring AMR.

**Table 3.** Concordance of AMR phenotype and genotypes Presence of resistance genes (G+) and number of phenotypically non-susceptible isolates (P+) are indicated. An asterisk indicates statistical significance by Fisher’s exact test; *P* value <0.05.

Antimicrobial agent	AMR gene	Characterization of phenotypic and genotypic resistance (*n*=36)				Concordance of phenotypic and genotypic resistance	
		P+/G+	P−/G+	P+/G−	P−/G−	OR (95 % CI)	***P*** value
GEN	*ant(6)-Ia*	8 (22.2 %)	7 (19.4 %)	16 (44.4 %)	5 (13.9 %)	0.36 (0.08–1.42)	0.175
	*aph(3')-IIIa*	–	5 (13.9 %)	24 (66.7 %)	7 (19.4 %)	0.06 (0.01–0.46)	0.010*
	*aadE*	3 (8.3 %)	1 (2.8 %)	21 (58.3 %)	11 (30.6 %)	1.57 (0.21–22.1)	>0.999
	*spw*	3 (8.3 %)	3 (8.3 %)	21 (58.3 %)	9 (25 %)	0.43 (0.09–2.17)	0.378
KAN	*ant(6)-Ia*	15 (41.7 %)	–	21 (58.3 %)	–	0.71 (0.04–14.4)	>0.999
	*aph(3')-IIIa*	5 (13.9 %)	–	31 (86.1 %)	–	0.16 (0.01–3.64)	0.294
	*aadE*	4 (11.1 %)	–	32 (88.9 %)	–	0.12 (0.01–2.94)	0.249
	*spw*	6 (16.7 %)	–	30 (83.3 %)	–	0.2 (0.01–4.40)	0.338
LIN, CLI	*lsa(E)*	6 (16.7 %)	–	30 (83.3 %)	–	0.20 (0.01–4.40)	0.338
	*lnu(B)*	6 (16.7 %)	–	30 (83.3 %)	–	0.20 (0.01–4.40)	0.338
	*lnu(C)*	1 (2.8 %)	–	35 (97.2 %)	–	0.03 (0–1.06)	0.104
ERY	*erm(A)*	2 (5.6 %)	–	28 (77.8 %)	6 (16.7 %)	0.43 (0.04–7.16)	0.476
	*erm(B)*	26 (72.2 %)	1 (2.8 %)	4 (11.1 %)	5 (13.9 %)	32.5 (3.79–390)	0.002*
	*erm(T)*	2 (5.6 %)	–	28 (77.8 %)	6 (16.7 %)	0.43 (0.04–7.16)	0.476
	*mef(A)*	6 (16.7 %)	1 (2.8 %)	24 (66.7 %)	5 (13.9 %)	1.25 (0.16–16.95)	>0.999
	*msr(D)*	5 (13.9 %)	1 (2.8 %)	25 (69.4 %)	5 (13.9 %)	1 (0.12–13.92)	>0.999
TET	*tet(O)*	23 (63.9 %)	–	12 (33.3 %)	1 (2.8 %)	1.91 (0.09–37.78)	>0.999
	*tet(W)*	6 (16.7 %)	–	29 (80.6 %)	1 (2.8 %)	0.21 (0.01–4.55)	0.347
	*tet(L)*	2 (5.6 %)	–	33 (91.7 %)	1 (2.8 %)	0.06 (0–1.70)	0.158
DOX	*tet(O)*	23 (63.9 %)	–	13 (36.1 %)	–	1.80 (0.09–34.88)	>0.999
	*tet(W)*	6 (16.7 %)	–	30 (83.3 %)	–	0.20 (0.01–4.40)	0.338
	*tet(L)*	2 (5.6 %)	–	34 (94.4 %)	–	0.06 (0–1.65)	0.154
OTC	*tet(O)*	23 (63.9 %)	–	11 (30.6 %)	2 (5.6 %)	4.18 (0.43–62.74)	0.278
	*tet(W)*	6 (16.7 %)	–	28 (77.8 %)	2 (5.6 %)	0.43 (0.04–7.16)	0.478
	*tet(L)*	2 (5.6 %)	–	32 (88.9 %)	2 (5.6 %)	0.12 (0.01–2.65)	0.230
LZD	*optrA*	–	2 (5.6 %)	–	34 (94.4 %)	17 (0.61–326.5)	0.154
CHL	*catA8*	1 (2.8 %)	–	19 (52.8 %)	16 (44.4 %)	0.84 (0.04–16.91)	>0.999
FLO	*catA8*	1 (2.8 %)	–	9 (25 %)	26 (72.2 %)	2.89 (0.14–56.62)	0.473

GEN, Gentamicin; KAN, kanamycin; LIN, lincomycin; CLI, clindamycin; ERY, erythromycin; TET, tetracycline; DOX, doxycycline; OTC, oxytetracycline; LZD, linezolid; CHL, chloramphenicol; FLO, florfenicol.

### Asian context

**Fig. 5.**
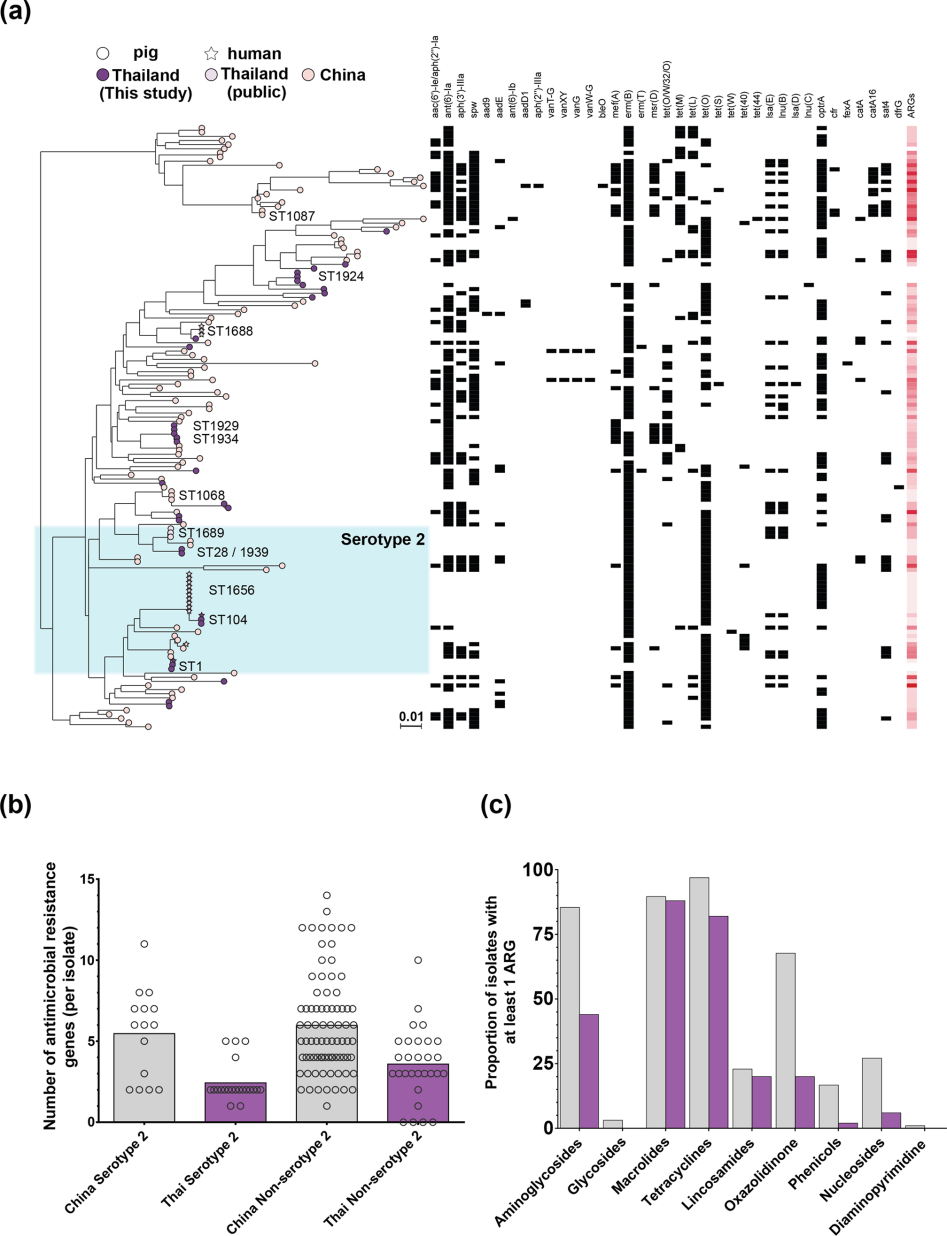
**(a) **Population structure of our Thai dataset compared to all public genomes from Asia in pubMLST (*n*=146; June 2022). All core genes (present in ≥95 % of isolates) from the reference genome BM407 (accession no. NC_012926.1) were used to build a gene-by-gene alignment. A maximum-likelihood phylogeny was reconstructed with iq-tree, using a GTR model and ultrafast bootstrapping (1000 bootstraps; version 1.6.8) [52, 120]. Bar, genetic distance of 0.01. Leaves are coloured by country of origin: Chinese isolates in pink; publicly available Thai isolates in light purple; and Thai isolates from this study in dark purple. Serotype 2 isolates are shaded in blue, with common sequence types (represented by two or more isolates) annotated. The presence of AMR genes identified using abricate and NCBI AMRfinder are indicated by black squares and increasing number of AMR genes by deeper red colour for each isolate. Interactive visualization is available on Microreact: https://microreact.org/project/ssuis-ns2-context [53]. (b) Smmary of the number of antimicrobial resistance (AMR) genes (ARG) contained, per isolate in Chinese (grey shading) and Thai (purple shading) serotype 2 and non-serotype 2 isolates. (c) Proportion of isolates that contain at least one ARG from each antimcrobial class.

For broader context, we compared our isolate genomes to all Asian isolate genomes shared publicly on pubMLST. While most public genomes were from Chinese pigs, it was clear that beyond the more studied serotype 2 isolates there remans a largely uncharacterized, diverse reservoir of *S. suis* diversity. Two separate clades of non-serotype 2 isolates can be observed in a maximum-likelihood phylogeny of the 112 publicly shared genomes and our collection (Fig. 5a, Table S8). While all isolates in our collection were predicted (and phenotypically) to have MDR, a large proportion of the publicly available Chinese isolates contained AMR determinants for even more drug classes. Overall, the Chinese isolates contained a greater number, and even more diverse collection of AMR genes (Fig. 5b, c).

## Discussion

*S. suis* were cultured and identified from 18.2 % of pigs swabbed in this study (138 of 760 samples), which is within the range previously reported for the prevalence in farmed pigs and slaughterhouses in the same area of Thailand [72, 73]. This level of prevalence was significantly lower than the *S. suis* prevalence previously reported in pigs from other provinces in Northern Thailand, such as Lampang (64.8 %) and Phayao (61.4 %) [33]. These and other studies in Northern Thailand reported a high prevalence of serotype 2 (5.6–43 %) and serotype 7 (8.2–14.3 %) isolates [72, 74]. However, in this study, we mainly identified non-serotype 2 isolates, with only a single isolate typed as serotype 9 and no serotype 2 isolates identified during this survey. This variation is likely due to differences in sampling, as we prioritized collection from healthy pigs. Invasive disease isolates have shown biogeographical variation, with competition and serotype replacement noted among virulent *S. suis* serotypes [17, 57, 75]. Serotype 9 is most common in diseased pigs from Europe and has a low pathogenic potential in humans, despite a rare case of serotype 9 infection in humans recently being reported in Thailand [9, 76].

Difficulties in capsule serotyping *S. suis* (*sensu lato*) isolates, where previously typed *S. suis* isolates are now designated as other *Streptococcus* species, hint at an ambiguous species designation and within-species diversity [77, 78]. This is further supported by characterization of divergent *S. suis* isolates by WGS [19]. The extent to which these represent stable lineages is unclear, with the rate of lineage turnover in *S. suis* seldom investigated [57, 79]. We identified increased variation in the core and accessory genomes of non-serotype 2 isolates (Figs 1 and 2). Serotype 2 isolates are typically found to have smaller genomes than non-invasive isolates and are often isolated from disease cases [12, 19]. Our collection of mostly non-invasive non-serotype 2 isolates had consistently larger genomes (with more genes; Table S2) than the *S. suis* reference genome (BM407 : 2 170 810 bp) and other invasive isolates (Table S2). Despite smaller genomes, these invasive isolates also tend to carry more virulence-related genes and all serotype 2 isolates in our collection carried the *pilB* gene, which is associated with the brain cell invasion required to cause meningitis in humans and pigs [80]. It has been suggested that this reduction in genome size may be due to gene loss, including core metabolism genes for nutrients that can be scavenged from the host; and a streamlining of functional/redundant elements [13, 81].

A plug-and-play theory of bacterial accessory genomes [82–84], where diversity in bacterial phenotypes can be conferred by a mobile pool of genes that are readily gained and lost, enables the acquisition of rapid adaptive genomic changes that can be spread through the population via recombination [58]. Host switching and zoonotic infection complicate analyses of gene flow and attribution of AMR elements [85, 86]. Here, we focus primarily on a potential reservoir of infection and characterize variation in the gene pool from which invasive disease isolates may have arisen. Where resistance is conferred by a single (or few) nucleotide substitution(s), it is impossible to tell from sequence data whether horizontal gene transfer (HGT) or point mutation was responsible [87, 88]. For other classes of antibiotics, the literature provides clear evidence for HGT of genes [27, 58, 89, 90]. For example, the pBM407 plasmid characterized in the pBM407 *S. suis* reference genome mobilizes *tet(O)*, *tet(L)*, *erm(B)*, *cat* and *dfr* genes between isolates [49, 91]. Including additional putative tetracycline-resistance genes, our analyses identified 18 accessory genes associated with resistance to seven antimicrobial classes.

We identified genes with described roles in resistance to aminoglycosides, macrolides, lincosamides, tetracycline, nucleoside, oxazolidinones and phenicols. Most isolates were predicted to have MDR (80.6 %; containing AMR determinants to three or more antibiotic classes). The most common AMR genes identified were associated with resistance to macrolides and tetracycline. More than 80 % of isolates contained at least one gene predicted to confer macrolide resistance [92]. The presence of *erm*(*B*) and *mef*(*A*) genes are consistent with previous studies, where *erm*(*B*) is strongly linked with macrolide-lincosamide-streptogramin B (MLS_B_) resistance and presented in 59–90 % of macrolide-resistant *S. suis* isolates from pigs [59, 93, 94]. The resistant gene *erm*(*T*) has been detected in *Streptococcus agalactiae *, *Streptococcus pyogenes* and other erythromycin-resistant isolates of group D streptococci [59, 95, 96], our identification of *erm*(*T*) in this study suggests potential within-genus HGT.

The most common tetracycline-resistance gene detected was *tet*(*O*) in over half of the isolates (63.9 %) (Table 3). An alternative ribosomal protein, *tet*(*M*), is also often associated with tetracycline resistance in *S. suis* [92, 97], but was not observed among our isolates. In addition, we detected *tet*(*L*) and *tet(W)* genes, which have not often been reported in *S. suis,* among non-serotype 2 isolates from healthy pigs. Corresponding phenotypic resistance to tetracycline was reported in over 90 % of isolates, which is consistent with global data reporting widespread resistance to tetracycline and macrolides, likely related to the prophylactic use in agriculture [60, 94, 96, 98]. AMR may play a role in increasing numbers of treatment failures [17, 96, 99], and in our study, despite widespread MDR, we observed phenotypic susceptibility to all three of the recommended antimicrobials used to treat clinical *S. suis* meningitis (penicillin, ceftiofur and ceftriaxone) [8, 61]. However, some β-lactam resistant isolates (18–27 %) were found among the non-clinical strains of *S. suis* [60, 96, 100] and β-lactam usage in pig production should be closely monitored, especially where there is prophylactic use in healthy pigs.

We report widespread phenotypic resistance, even in the absence of a predictive resistance element (Fig. 3). Given the enhanced genetic diversity and lack of clear characterization of this disease reservoir, it is possible that additional resistance elements have yet to be fully described. A recent study by Hadjirin *et al.* identified more than 20 novel *S. suis* AMR determinants, including point mutations that have not been included in this study [101]. The authors also note poorer corelation between genotype and phenotype for fluoroquinolones and phenicols, complex interactions between AMR determinants – with some AMR genes able to confer resistance to multiple classes of antimicrobial. Even in the absence of direct antimicrobial selective pressure, broad spectrum use of antibiotics acts on all bacterial species in the microbiome; and this bystander effect can confer resistance on bacterial species that are not the target of the antimicrobial treatment [51, 102]. Enrofloxacin is widely used to treat other types of bacterial infection in the respiratory and digestive systems of livestock animals, and in our collection more than 40 % of isolates were resistant to this antibiotic [96, 103]. Spectinomycin is often used in pig production and other livestock animals combined with lincomycin [104, 105]. Clusters of AMR genes [*aadE-spw-lsa(E)-lnu(B)*] have been identified in staphylococci and enterococci associated with lincosamide resistance [106, 107]. We identified this combination of spectinomycin and lincosamide resistance in one serotype 2 isolate and two non-serotype 2 isolates from healthy pigs. Individually, we identified spectinomycin and lincosamide resistance genes in a small number of isolates, as has previously been reported for invasive *S. suis* isolates [97, 108].

The plasmid-borne chloramphenicol-resistance gene, *catA8* [109, 110], *and* the *optrA* gene that confers transferable combined resistance to oxazolidinones (linezolid) and phenicols (chloramphenicol and florfenicol) [111–113], are reported here for the first time, to our knowledge, for *S. suis* in Thailand. Although phenotypic susceptibility was recorded to linezolid, the isolates were resistant to chloramphenicol and florfenicol. Recently, *optrA* has been found in oxazolidinone-resistant *S. suis* isolates in China [114–116]. This is further evidence of the unintended effect of broad-spectrum antimicrobials, such as the oxazolidinones linezolid and tedizolid, which are highly effective against Gram-positive bacteria [117] but rarely used in the pig production industry. However, florfenicol has been used in livestock animals for therapeutic purposes and there is documented transfer of plasmids carrying *optrA* between different Gram-positive bacteria [118]. Twenty-one different resistance gene patterns were observed, with *erm*(*B)* and *tet*(*O*) found together in 62.5 % (5 of 8) of serotype 2 isolates, as previously observed [108]. Most non-serotype 2 isolates possessed AMR genes to at least three antimicrobial classes (up to seven; 22/28, 78.6 %). Several genetic elements, including ICEs carrying AMR genes such as *optrA*, *erm(B)*, *tet(M)*, *tet(O)* and *tet(W)*, have been reported in *S. suis* [49, 108]; however, plasmid elements were found only in non-serotype 2 isolates in this study.

In line with other studies, we also observed differences in the extent of AMR between countries. Isolates from China were more extensively drug resistant than isolates from Chiang Mai and Thailand. Differences have previously been observed between the UK and Canada, potentially linked to differences in veterinary antimicrobial usage [119]. Genome comparisons between Chinese and Thai isolates also highlight separate reservoir gene pools, with long branches of non-invasive isolates (Fig. 5). This hints at large, uncharacterized reservoirs of diversity in healthy pigs around the world.

### Conclusion

We collected isolates from 760 healthy pigs reared in the pork industry in Northern Thailand. Through comparison of 36 whole-genome sequences, we identified increased genetic diversity in these non-serotype 2 carriage isolates, from which the more invasive and pathogenic serotype 2 isolates emerge. Corresponding diversity was also seen in the breadth and diversity of AMR determinants that conferred increased phenotypic non-susceptibility. This genetically diverse reservoir of *S. suis* pose a public-health risk with the potential for gene flow to more invasive isolates, broadening their spectrum of AMR. Extensive phenotypic resistance is observed to antimicrobials that are not typically used to treat this infection. This can be partly explained by the co-occurrence of resistance genes on ICEs. However, little phenotypic resistance was observed to β-lactams, which remain the prescribed antimicrobial for *S. suis* infection in Thailand. Continued surveillance and more stringent control of antimicrobial usage within the pork industry will be necessary to monitor a growing AMR threat in *S. suis.*

## Supplementary Data

Supplementary material 1

Supplementary material 2

Supplementary material 3

Supplementary material 4

Supplementary material 5

Supplementary material 6

Supplementary material 7

Supplementary material 8
